# Regulation of mating type switching by the mating type genes and *RME1* in *Ogataea polymorpha*

**DOI:** 10.1038/s41598-017-16284-7

**Published:** 2017-11-24

**Authors:** Katsuyoshi Yamamoto, Thi N. M. Tran, Kaoru Takegawa, Yoshinobu Kaneko, Hiromi Maekawa

**Affiliations:** 1 Graduate School of Engineering, Osaka University, Osaka, Japan; 2 Faculty of Agriculture, Kyushu University, Fukuoka, Japan; 3 Centre for Promotion of International Education and Research, Faculty of Agriculture, Kyushu University, Fukuoka, Japan

## Abstract

*Saccharomyces cerevisiae* and its closely related yeasts undergo mating type switching by replacing DNA sequences at the active mating type locus (*MAT*) with one of two silent mating type cassettes. Recently, a novel mode of mating type switching was reported in methylotrophic yeast, including *Ogataea polymorpha*, which utilizes chromosomal recombination between inverted-repeat sequences flanking two *MAT* loci. The inversion is highly regulated and occurs only when two requirements are met: haploidy and nutritional starvation. However, links between this information and the mechanism associated with mating type switching are not understood. Here we investigated the roles of transcription factors involved in yeast sexual development, such as mating type genes and the conserved zinc finger protein Rme1. We found that co-presence of mating type **a**1 and *α*2 genes was sufficient to prevent mating type switching, suggesting that ploidy information resides solely in the mating type locus. Additionally, *RME1* deletion resulted in a reduced rate of switching, and ectopic expression of *O. polymorpha RME1* overrode the requirement for starvation to induce *MAT* inversion. These results suggested that mating type switching in *O. polymorpha* is likely regulated by two distinct transcriptional programs that are linked to the ploidy and transmission of the starvation signal.

## Introduction

Similar to many other organisms, yeast senses and engages appropriate response programs following changes in their environment, including invasive growth, dimorphic transitions, and sexual differentiation. Spore formation, which is often accompanied by mating/meiosis, is a strategy for survival in harsh environments in many yeast species. Homothallism, which is a characteristics observed in some yeasts, allows mating to occur in populations derived from a single cell and is achieved by switching mating type frequently during vegetative growth in *Saccharomyces cerevisiae* ^1^. Mating type in *S. cerevisiae* is determined by two nonhomologous alleles in a single mating type locus (*MAT*), *MAT* **a** or *MATα* ^1,2^. Each of these sequences encodes transcriptional regulators of the two different haploid mating types. Mating type switching is mediated by a site-specific homologous recombination event that replaces one *MAT* allele with silent DNA sequences, *HML* **a** or *HMR*α which encodes the opposite *MAT* allele that is located on the same chromosome. A similar mechanism comprising three mating type gene sequences and the site-specific homothallic switching (HO) endonuclease, which generates a double-strand break, are shared among species closely related to *S. cerevisiae* such as *Candida glabrata*, *Zygosaccharomyces rouxii*, and *Kluyveromyces lactis* ^3,4^. Interestingly, the *HO* gene in *K. lactis* is non-functional, and new mechanisms inducing DNA double-strand breaks have been acquired during evolution^5,6^. In *S. cerevisiae*, expression of the *HO* gene is a trigger to induce mating type switching^1^. Recently, a novel mode of mating type switching was reported in methylotrophic yeast, including *Ogataea polymorpha*, which utilizes a chromosomal recombination between inverted-repeat sequences flanking two *MAT* loci^7,8^. The inversion is highly regulated and occurs only when cells are haploid and under starvation conditions. However, the links between this information and the mechanism associated with mating type switching remain unknown.

Changes in transcriptional profile underlie the molecular mechanisms that induce mating/meiosis and sporulation in yeast. Genes specifically expressed in **a** or α cells, namely haploid-specific genes (hsgs), are essential for inducing the mating process, whereas they are repressed in situations where both mating type alleles are present in a single cell (e.g., meiosis-competent diploid cells)^1,2^. Many hsgs and their expression patterns appear to be common among ascomycetous yeasts, many of which are components of the mating pheromone-signalling pathway^9^; however, there may exist differences in the regulatory mechanism^9^. In *S. cerevisiae*, hsgs are repressed by a heterodimer of homeodomain proteins **a**1 and α2, whereas *K. lactis* hsgs are placed under the direct regulation of Rme1, a conserved zinc finger transcription factor. Unlike *S. cerevisiae*, *K. lactis* primarily proliferate as haploid, and mating type switching and subsequent mating are tightly connected, both of which occur only under starvation conditions that lead to the spore formation. The starvation signal is incorporated into these programs through induction of the expression of *RME1*. Additionally, in *K. lactis*, **a**1-α2 is involved in the regulation of hsgs expression through repressing *RME1*. Rme1 was originally identified as a regulator of mating type switching and it directly binds to *MAT* as well as activates transcription of the *KAT1* gene which encodes a domesticated transposase^6,10^.

Recognition of mating partners is initiated when the pheromone receptor binds to the mating pheromone secreted from cells of the opposite mating type^11,12^. In the model systems, *S. cerevisiae* and the fission yeast *Schizosaccharomyces pombe*, pheromones and pheromone receptor genes are targets of transcriptional regulation by pheromone signalling, which is transmitted through a heterotrimeric G protein and the downstream MAP kinase pathway to ensures the robustness of the signalling and the engagement of cells to the mating process^13–15^. Many of these genes are transcriptionally induced by starvation in yeast species, such as *S. pombe*, *K. lactis*, and *Candida lusitaniae*, where sexual development is restricted under starvation conditions^16–19^.

Here, we evaluated the roles of transcription factors involved in sexual development in methylotrophic yeast *Ogataea polymorpha*, including mating type genes and a conserved transcription factor, Rme1, whose orthologue in *K. lactis* is involved in mating type switching^6,10^. Our results indicated that co-expression of **a** *1* gene in *MAT* **a** and *α*2 gene in *MATα* were sufficient to prevent mating type switching, suggesting that the combination of mating type genes generates ploidy information for mating type switching. Furthermore, *RME1* deletion resulted in reduced mating type switching, whereas its ectopic expression overrode the requirement for starvation for inducing *MAT* inversion. There results suggested that mating type switching in *O. polymorpha* is likely regulated by **a**1-α2 and Rme1.

## Results

### Mating type genes are not required for the *MAT* inversion

**Figure 1:**
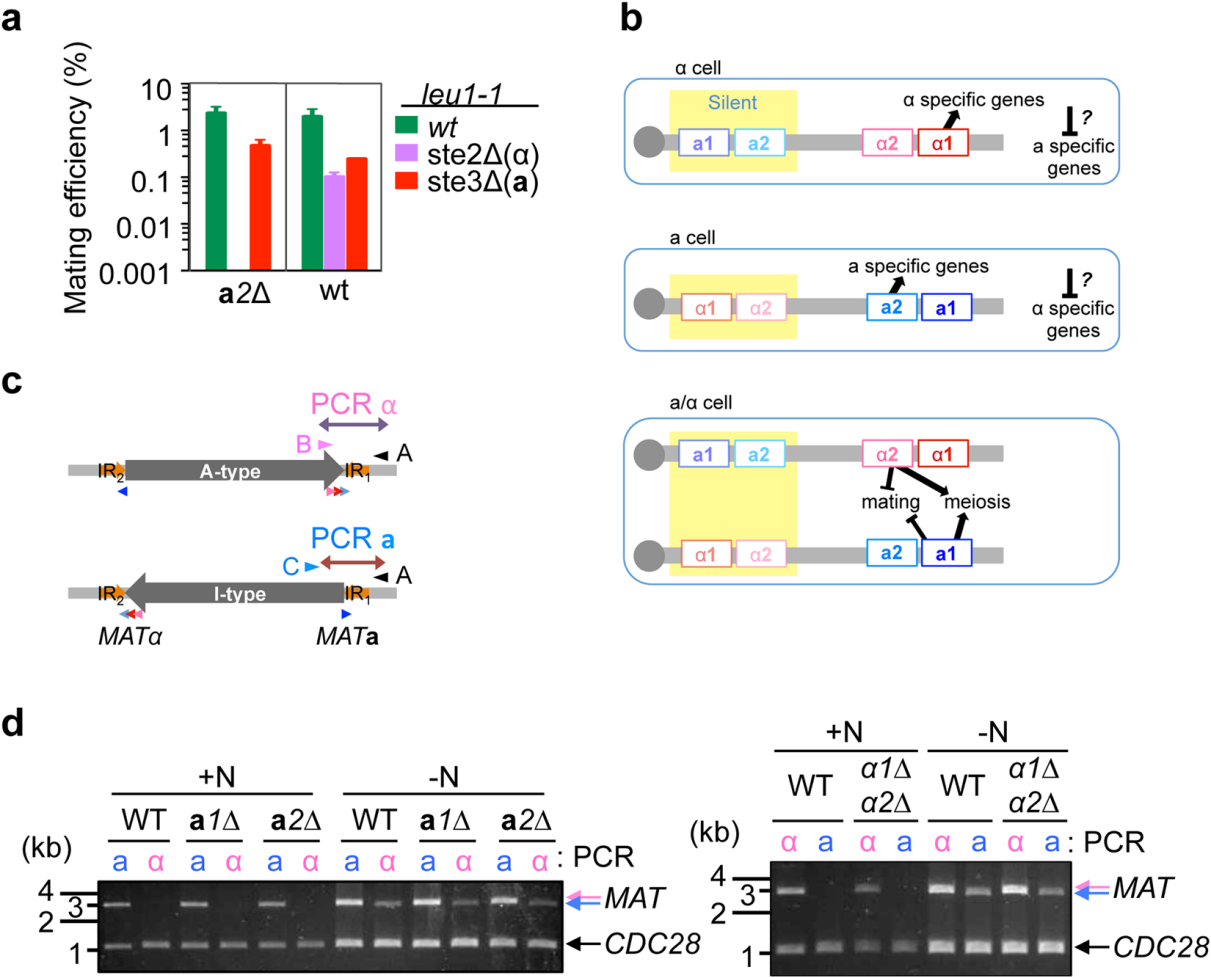
Mating type genes are not required for the *MAT* inversion. (**a**) **a** *2* gene is required for mating with α cell but not with **a** cell. Wild-type and **a** *2*∆ strains of *ura3–1* (BY4330 and HPH922, respectively) and wild-type, *ste2*∆ and *ste3*∆ strains of *leu1–1* (HPH22, HPH553, and HPH581, respectively) genotypes were combined on MEMA mating medium and incubated at 30 °C. After 24 h, cells were spread on SD plates to select for Leu^+^Ura^+^ diploids. Colony number was counted after 2-day incubation at 37 °C. The average of three independent crosses is shown. Error bars indicate SD. (**b**) Function of mating type genes in establishing mating type identity. Grey circle represents centromere^7^. (**c**) Schematic of the primer designs for PCR reactions specific to I(**a**)- or A(α)-type chromosomes (*MAT* PCR analysis). (**d**) I(**a**)- or A(α)-type of *MAT* chromosome was determined by PCR reactions described in (**c**). PCR reactions to detect I(**a**)- or A(α)-type chromosomes are designated as **a** and α and designated a Genomic DNA samples were prepared from the wild-type strain (HPH1047 and HPH1050) and deletion mutants for *MAT* **a**1, *MAT* **a**2, or *MATα* (HPK073, HPH1255, and HPK072) before (+N) and after incubation on MEMA medium (−N). *CDC28*-specific primer set was added to all PCR reactions as controls for the amount of input DNA. Full-length gels are presented in Supplementary Figure S5.

We previously reported α1 is essential for conjugation of α cells with **a** cells^8^. Because Hanson *et al*.^7^ reported the expression of an **a**2-like gene in *O. polymorpha*, we sequenced **a**2 mRNA and revealed that a predicted intron was indeed removed from mRNA to encode an **a**2 like protein. In order to examine a possible role of **a**2 in mating, we crossed **a**2∆ cells with deletion mutants of α-factor and **a**-factor receptor genes *STE2* and *STE3*, respectively. Because *ste2*∆ cells cannot respond to α-factor, homothallic *ste2*∆ cells are able to mate with cells expressing **a** identity. Similarly, *ste3*∆ cells can mate only with α cell. In our mating assay, while **a** *2*∆ cells were able to mate with *ste3*∆ cells, no mating was observed when crossed with *ste2*∆ cells (Fig. 1a). This was the reversed pattern of mating capability in *α*1∆ cells^8^. These data indicated that *O. polymorpha* required **a**2 and α1 for **a**-, and α-cell identity, respectively (Fig. 1b). Consistently, both **a** *2* and α1 genes were expressed in mitotically growing **a** and α cells respectively as well as those cells under starvation condition (Supplementary Fig. S1a).

Analysis of RNA expression level revealed that all four mating type genes were expressed mitotically and induced upon starvation (Supplementary Fig. S1). We therefore examined whether *MAT* gene products contributed to the efficient mating type switching. We observed that mating type switching was not reduced in cells harbouring deletions of each mating type genes (Fig 1c,d), suggesting that *MAT* genes did not play important roles in the mating type switching.

### *MAT* inversion is inhibited in meiosis-competent diploid cells

**Figure 2:**
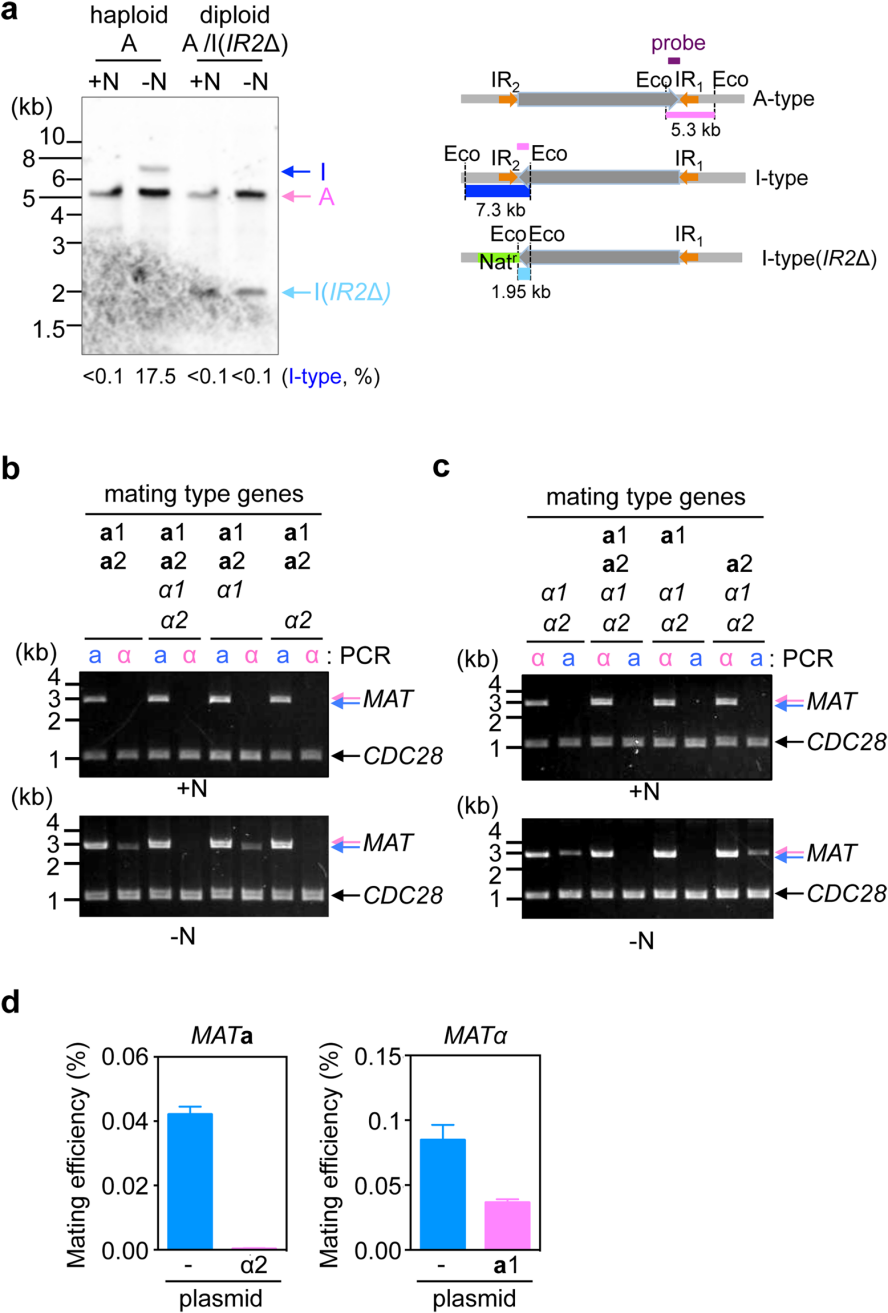
**a**1-α2 inhibits the *MAT* inversion. (**a**) *MATα* haploid cells (HPH848) and *MATα*/*MAT* **a**(IR2∆) diploid cells (HPH964) were grown in YPDS medium (+N) and transferred to MEMA medium and incubated for 18 hrs (−N). Genomic DNA samples were prepared and digested with EcoRI and subjected to Southern blot analysis using a probe specific for *MATα* sequence (Left panel). The percentage of I-type chromosomes were calculated by measuring the intensity of A- and I-type bands and presented below the blot. Right panel: schematics of *MAT*-containing chromosomes with the indicated genotype and predicted fragment sizes for the probe (purple) in Southern blot analysis. (**b** and **c**) Genomic DNA was prepared from strains with the indicated genotype (HPH1047, HPH1050, HPH1201, HPK011, HPK084, HPK085, HPK092, and HPK093), and the mating type was determined by *MAT* PCR analysis as shown in Fig. 1c. +N: cells grown in YPDS medium, −N: cells incubated in MEMA medium for 16 hrs. *CDC28*-specific primer set was added to all PCR reactions as controls for the amount of input DNA. (**d**) *MAT* **a** *IR2*∆ haploid strain with *ura3-1 ade12-cr3* genotype (HPH1174) and the same strain carrying pHM961 expressing *α2* gene (HPH1702) were crossed with wild type strain (HPH22) on NaKG mating medium and incubated at 30 °C. Similarly, *MATα IR2*∆ haploid strain with *ura3-1 leu1-1* genotype (HPH1162) and the same strain carrying pHM964 expressing **a** *1* gene (HPH1699) were crossed with wild type strain (BY4331). After 24 h, cells were spread on SD + Ura plates to select for Leu^+^Ade^+^ diploids. Colony number was counted after 2-days incubation at 37 °C. The average of three independent matings is shown. Error bars indicate SD. Full-length blot and gels are presented in Supplementary Figure 5.

Two sexual differentiation programs, mating and meiosis, are induced by starvation in *O. polymorpha* ^20^. Importantly, the mating program should become active only in **a-** or α- haploid cells and is repressed in **a**/α diploid cells, whereas initiation of meiosis requires both *MAT* **a** and *MATα* gene products, which makes mating and meiosis mutually exclusive^8^. Because mating type switching could potentially reduce meiosis efficiency, it might be inhibited in **a**/α diploid cells. We examined the inversion of the *MAT* intervening region (*MAT* inversion) in the A(α)-type chromosome in α haploid and **a**/α haploid cells. Because it is impossible to distinguish original **a**/α from α/**a** diploid cells generated by inverting the *MAT*-intervening region on both chromosomes, we deleted the centromere proximal inverted repeat (IR2) of the I(**a)**-type chromosome to prevent the inversion and to obtain a DNA fragment of different size from wild type I(**a)**-type chromosome in Southern blot analysis (Fig. 2a)^8^. Inversion efficiency was calculated at ~17.5% in haploid cells, but was below detectable level in **a**(*IR2*∆)/α-diploid cells.

The inhibition of the inversion might have occurred due to the co-presence of the two *MAT* alleles, rather than ploidy. To investigate this possibility, we introduced a *MATα* allele at the *URA3* locus in **a** cells. As expected, **a** cells expressing *MATα* were unable to undergo the *MAT* inversion (Fig. 2b). Additionally, the *MATα*(*α2*∆) plasmid was unable to inhibit the *MAT* inversion in **a** cells, whereas the *MATα*(*α*1∆) plasmid inhibited the inversion to the same degree as that observed by the *MATα* plasmid. We also performed the same experiment in α haploid cells, finding that expression of the **a**1 gene inhibited the *MAT* inversion in α haploid cells (Fig. 2c). These results suggested that co-expression of **a**1 and *α*2 was sufficient to prevent activation of the mating type switching.

To further investigate the effect of co-expression of **a** *1* and *α*2 genes, *α2* gene was expressed exogenously in stable **a** cells (mating type of the strain is unswitchable because of the deletion of IR2 sequences) and crossed with wild type cells. The mating efficiency was severely reduced compared to the parental stable **a** cells. The equivalent experiment was performed in stable *α* cells with a similar result, although the extent of the mating inhibition was much weaker (Fig. 2d). These results suggested that co-expression of **a**1 and α2 had inhibitory effect on mating.

### Mating type switching occurs following cessation of mitotic cell division upon nutrient starvation in *O. polymorpha*

**Figure 3:**
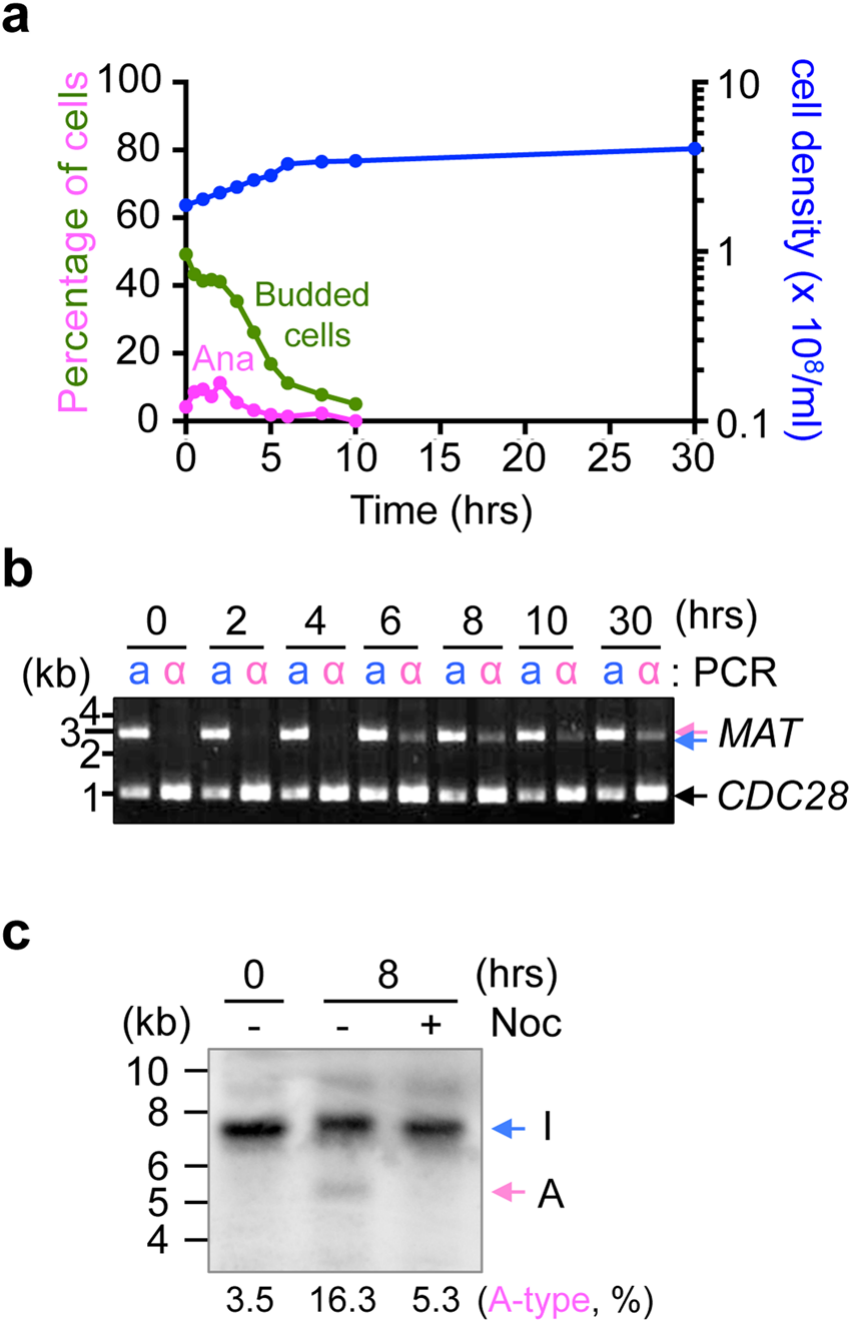
Mating type switching occurs after cessation of cell division under starvation condition. (**a**) Wild type CBS4329 cells were grown in YPDS medium and transferred to NaKG medium. Samples were collected at different intervals and cell density was measured using a hematocytometer. The remaining of samples were fixed and stained with DAPI to determine anaphase cells (Ana, indicated by magenta) and budding index (Budded cells, indicated by green). (**b**) Genomic DNA was prepared from samples collected in (**a**) and subjected to *MAT* PCR analysis as shown in Fig. 1c. *CDC28*-specific primers were added to PCR reactions as controls for the amount of input DNA. (**c**) I(**a**)-type of CBS4329 cells grown in YPDS medium were incubated in NaKG medium containing either dimethyl sulfoxide or nocodazole for 8 hrs. Genomic DNA samples were prepared and digested with EcoRI and subjected to Southern blot analysis using the probe specific for *MATα* sequences shown in Fig. 2a. The percentage of A-type chromosomes was calculated by measuring the intensity of A- and I-type bands and presented below the blot. Full-length gels are presented in Supplementary Figure S5.

To better understand the physiological conditions that allow the mating type switching to occur in *O. polymorpha*, the timing of the *MAT* inversion was investigated more precisely (Fig. 3). Wild type cells grown in yeast extract, peptone, and dextrose medium containing 200 mg/L adenine, leucine, and uracil (YPDS) until the late log phase [optical density at 663 nm (A663) = 10–15] were transferred to minimal medium without nitrogen (NaKG; 0.5% sodium acetate, 1% potassium chloride, and 1% glucose). We observed that the percentage of budded cells remained relatively high (40–45%) over the course of 2.5 h, followed by a gradual decrease to 5% after 10 h following the shift (Fig. 3a). An inverted type of chromosome was first detected at the 6 hr time point when budded cells were dropped and almost no anaphase cells were observed (Fig. 3b). The ratio of inverted chromosomes did not increase to a large degree over the next 24 hrs as judged by the intensity of polymerase chain reaction (PCR) products (Fig. 3b). These results suggested that the chromosomal inversion of the *MAT*-containing region occurred as cells ceased mitosis. When nocodazole was added into the culture at the time of the shift to NaKG medium, the inverted ratio became lower than that observed in the DMSO control cells (Fig. 3c). This might indicate that the starvation signal by itself was insufficient to induce the *MAT* inversion and that cell cycle progression was required.

### Pheromone signalling does not induce mating type switching

**Figure 4:**
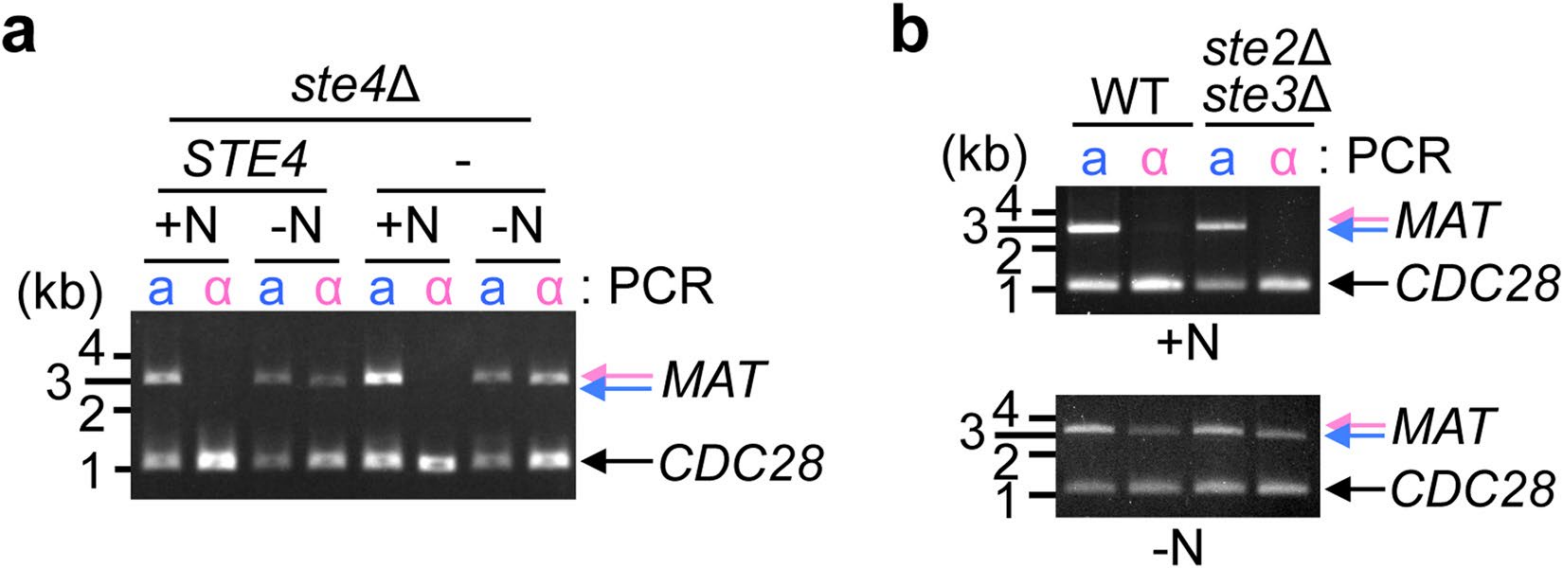
The pheromone signalling pathway is not required for the *MAT* inversion. (**a**) *Ste4*∆ cells and *ste4*∆ cells carrying the *STE4* plasmid (HPH1268 and HPH1267, respectively) grown in YPDS medium were incubated in NaKG medium for 12 hrs. Genomic DNA was subjected to *MAT* PCR analysis as shown in Fig. 1c. *CDC28*-specific primers were added to PCR reactions as controls for the amount of input DNA. (**b**) *STE2 STE3 ku80*∆ (HPH1379) and *ste2*∆ *ste3*∆ *ku80*∆ cells (HPH1696) grown in YPDS medium were incubated in NaKG medium for 12 hrs. Genomic DNA was subjected to *MAT* PCR analysis as shown in Fig. 1c. *CDC28*-specific primers were added to PCR reactions as controls for the amount of input DNA. Full-length gels are presented in Supplementary Figure S5.

We then investigated the transcriptional programs activated during nutrient starvation. In *O. polymorpha*, the pheromone signalling pathway is likely activated under starvation condition because some of pheromone signalling components, such as *STE4* and *STE18* were transcriptionally induced in malt-extract/maltose mating medium (MEMA) (Supplementary Fig. S2). We found that *ste4*∆ cells were sterile as predicted, but that the *MAT* inversion occurred as did in wild-type cells (Fig. 4a). Furthermore, *ste2*∆ *ste3*∆ cells, that lacks both **a**- and α-factor receptors, underwent the *MAT* inversion (Fig. 4b). Therefore, our results indicated that the mating type switching did not require the activation of the pheromone signalling pathway.

### The transcription factor Rme1 is required for efficient inversion and sexual differentiation

**Figure 5:**
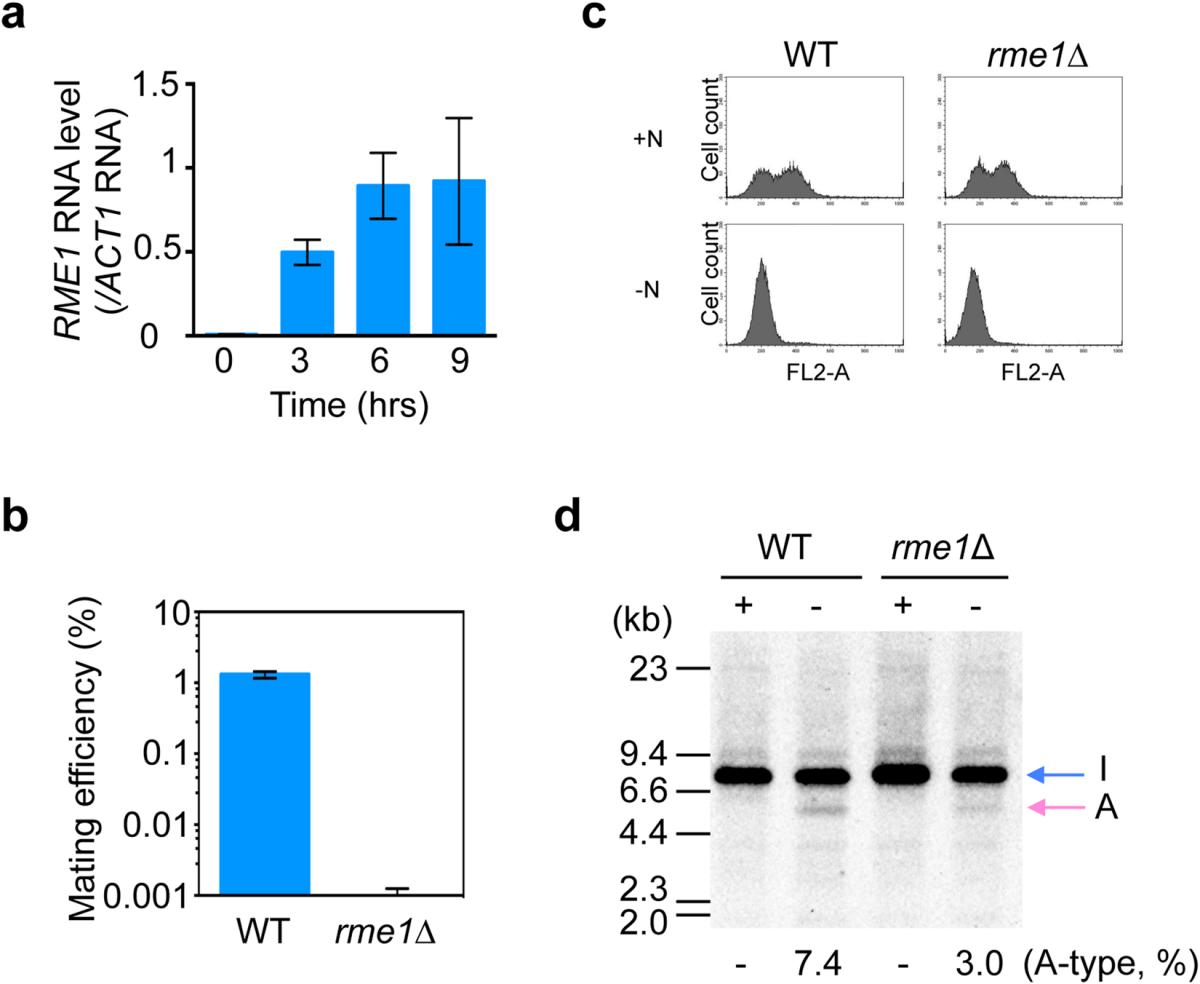
Rme1 is required for mating and *MAT* inversion. (**a**) *RME1* mRNA level increases in response to nutritional starvation. The qPCR analysis was performed with RNAs prepared from wild-type cells grown in YPDS and from the same cells following incubation in NaKG medium. RNA levels were normalized to that of *ACT1* RNA. Shown are the averages of three independent PCR reactions. Error bars indicate SD. (**b**) Mating is severely reduced in *rme1*∆ cells. Wild-type and *rme1∆* strains with *ura3-1* genotypes (HPH47 and HPK021, respectively) were combined with wild type cells with *leu1-1* genotype (HPH22) on NaKG mating medium and incubated at 30 °C. After 30 h, cells were spread on SD plates to select for Leu^+^Ura^+^ diploids. Colony number was counted after 2-days incubation at 37 °C. The average of three independent matings is shown. Error bars indicate SD. (**c**) Wild type (BY21401) and *rme1*∆ (HPK187) were grown in YPDS medium and shifted to NaKG medium and incubated for 12 hrs. Cells were fixed with 70% ethanol and DNA content was measured by flowcytometry. (**d**) *MAT* inversion was reduced in *rme1*∆. Genomic DNA was prepared from strains with the indicated genotype (HPK161 and HPK163), digested with EcoRI, and subjected to Southern blot analysis as shown in Fig. 2a. +N: cells grown in YPDS medium, −N: incubated in NaKG medium for 15 hrs. Full-length gels are presented in Supplementary Figure S5.

Rme1 is a transcription factor which plays important roles in sexual differentiation in yeast. A homolog of *RME1* gene (*OpRME1*) was found in the *O. polymorpha* genome. Because the expression of *OpRME1* gene was strongly induced upon starvation (Fig. 5a), the roles in sexual cycle were investigated. Deletion of *OpRME1* did not completely abolish mating capability, because we were able to backcross the original *rme1*∆ isolates with a wild-type strain. However, when crossed with wild type cells, *rme1*∆ cells exhibited >1000-fold less mating efficiency as compared with wild-type cells (Fig. 5b and Supplementary Fig. S3), suggesting that Rme1 was required for mating. We also examined cell cycle arrest upon starvation in *rme1*∆ cells because G1 arrest is the prerequisite for yeast mating (Fig. 5c). The *rme1*∆ cells arrested with 1C DNA content as observed in wild type cells, suggesting Rme1 is dispensable for arresting cell cycle in G1. We also examined *MAT* inversion following starvation and found that it was >50% less frequent compared with that observed in wild-type cells (Figs. 5d, 7.4% of inverted orientation in wild type cells vs. 3.0% in *rme1*∆ cells). These results suggested that Rme1 played important roles in inducing mating type switching and mating.

**Figure 6:**
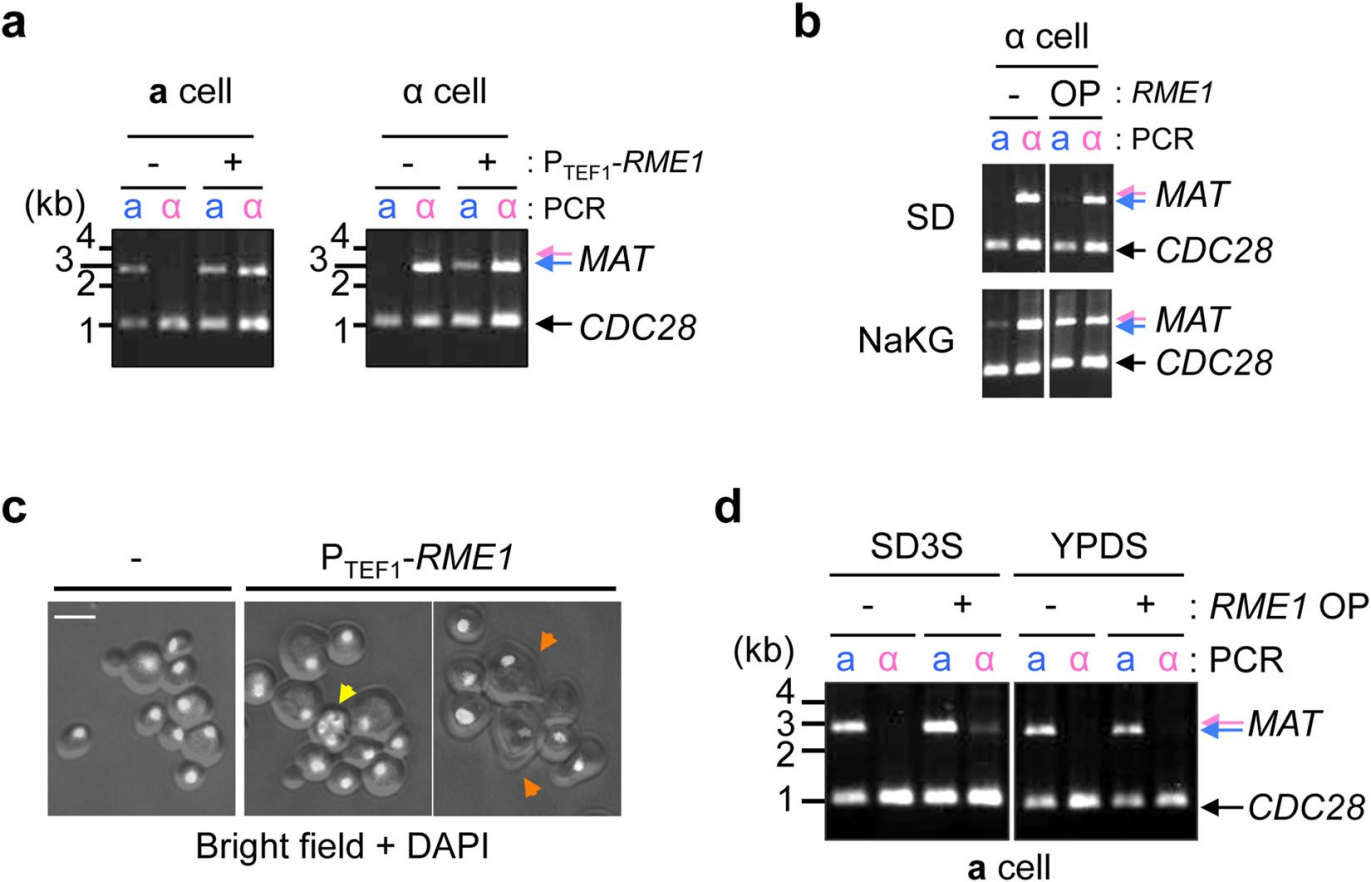
*RME1* overexpression induces the *MAT* inversion in mitotically growing cells. (**a**) *RME1* was expressed in I(**a**)- and A(α)-type wild-type strains (CBS4329) using a strong constitutive *TEF1* promoter. Cells were grown in SD medium supplemented with adenine, leucine, and uracil, and genomic DNA was subjected to *MAT* PCR analysis as shown in Fig. 1c. (**b**) A(α)-type wild-type cells carrying P_TEF1_-*RME1* were grown in SD medium (+N), transferred to NaKG medium and incubated for 20 hrs (−N). Genomic DNA was subjected to *MAT* PCR analysis as shown in Fig. 1c. *CDC28*-specific primers were included in the reaction as control for input DNA. RME1 -: cells carrying vector (pHM874), OP: cells carrying P_TEF1_-*RME1* (pHM960). (**c**) Wild-type cells carrying vector (pHM874) or P_TEF1_-*RME1* (pHM960) (OPT1 and OPT2, respectively) were grown on SD plate supplemented with adenine, leucine, and uracil for 3 days at 30 °C. Cells were fixed with ethanol and stained with DAPI. Mating projection-like morphology (orange arrows) and meiotic nuclei (yellow arrow) were evident only in cells carrying P_TEF1_-*RME1*. (**d**) I(**a**)-type wild type cells (HPH466) carrying either vector (pHM874) or P_TEF1_-*RME1* (pHM960) were grown in SD medium supplemented with adenine, leucine, and uracil or YPDS medium. Genomic DNA was subjected to *MAT* PCR analysis as shown in Fig. 1c. *CDC28*-specific primers were included in the reaction as control for input DNA. Full-length gels are presented in Supplementary Figure S5.

To further confirm Rme1’s involvement in mating type switching, *RME1* was expressed in mitotically growing cells from a strong constitutive *TEF1* promoter in *O. polymorpha* (Fig. 6). *RME1*-overexpressing cells grown in synthetic defined (SD) medium were a mixture of A(α) and I(**a**) types, whereas wild-type cells maintained their original *MAT* chromosome type, indicating that *MAT* inversion occurred in *RME1*-overexpressing cells in both A-to-I and I-to-A directions (Fig. 6a). These results suggested that *RME1* overexpression was capable of overriding the requirement for nutritional starvation to induce *MAT* inversion. Moreover, *MAT* inversion was further induced in *RME1*-overexpressing cells in starvation medium to a level higher than that observed in wild-type cells under the same condition (Fig. 6b). These results suggested that *RME1* expression alone was insufficient for inducing mating type switching, but it might constitute a rate-limiting factor. Moreover, *RME1*-overexpressing cells exhibited morphological changes that resembled mating projections, as well as signs of meiosis and sporulation (Fig. 6c). Therefore, our findings indicated that *RME1* overexpression activated most, if not all, biological programs in response to nutritional starvation, including mating type switching.

Notably, the effect of *RME1*-overexpression on *MAT* inversion was not evident in YPDS medium (Fig. 6d). In *K. lactis*, Rme1 protein levels were 7-fold higher in cells grown in synthetic complete medium as compared with levels observed in cells grown in YPD medium^6^. Therefore, Rme1 expression might be similarly regulated in *O. polymorpha*.

### *MAT* inversion is autophagy dependent

**Figure 7:**
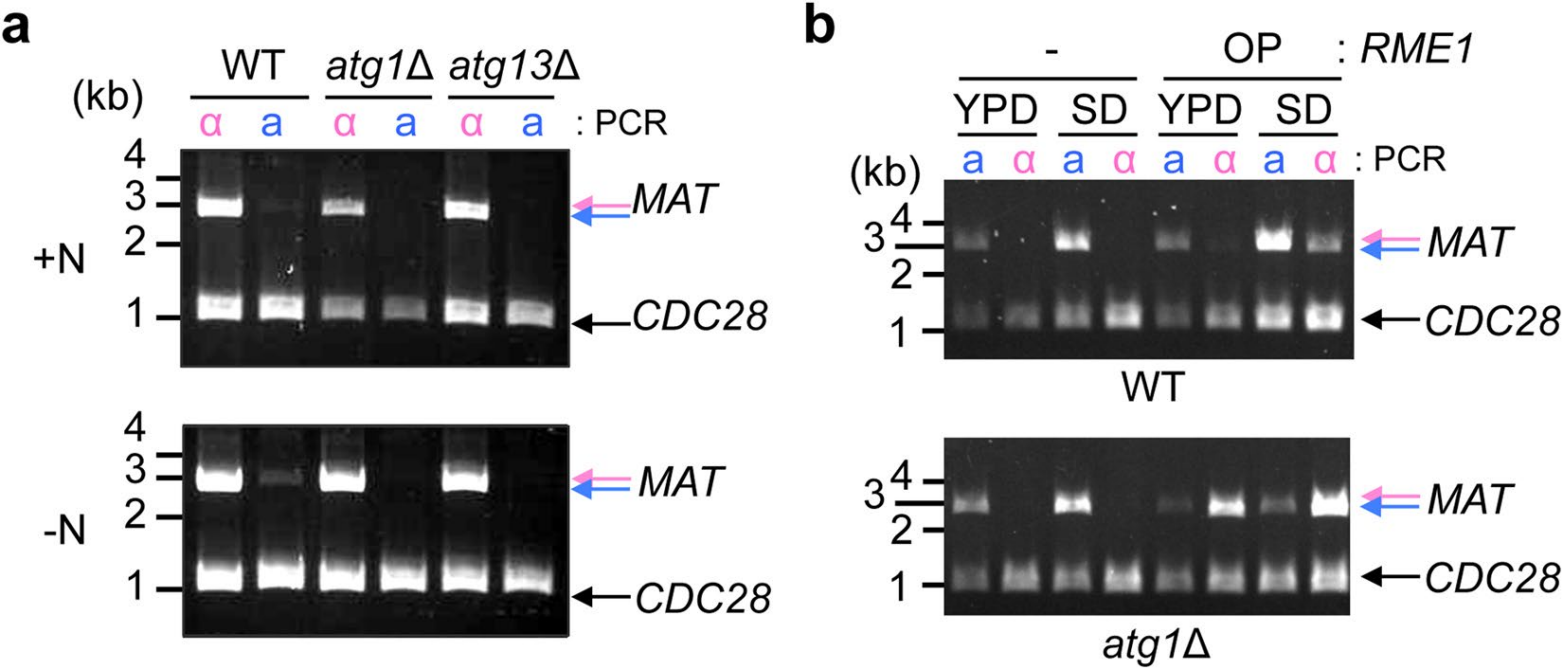
*MAT* inversion is autophagy dependent. (**a**) Autophagy mutants are *MAT* inversion deficient. Wild-type, *atg1*∆, and *atg13*∆ cells (HPH848, HPH1561, and HPH1556, respectively) were grown in YPDS medium (+N), transferred to MEMA medium and incubated for 15 hrs (−N). Genomic DNA was subjected to *MAT* PCR analysis as shown in Fig. 1c. (**b**) Atg1 is dispensable for *REM1*-induced *MAT* inversion. Wild-type (BY2140) and *atg1*∆ cells (HPH1620) carrying either vector (pHM874) or P_TEF1_-*RME1* (pHM960) were grown in YPDS or SD medium supplemented with adenine, leucine, and uracil. Genomic DNA was prepared and subjected to *MAT* PCR analysis as shown in Fig. 1c. *CDC28*-specific primers were included in the reaction as control for input DNA. Full-length gels are presented in Supplementary Figure S5.

Important physiological changes caused by nutritional starvation involve the induction of autophagy. Protein degradation by autophagy provides necessary materials for the cellular response to nutritional starvation, and therefore deletion mutants of core autophagy factors such as *ATG1* results in a severe defect in meiosis and sporulation in both *S. cerevisiae* and *S. pombe* ^21–23^. Because the mating type switching in *O. polymorpha* is induced by nutritional starvation, we examined involvement of autophagy in the mating type switching. We found that the *MAT*-inversion was reduced in *atg1*∆ and *atg13*∆ cells, suggesting that the mating type switching is an event downstream of autophagy (Fig. 7a).

*RME1* transcription was induced in *atg1*∆ cells as well as in wild type cells upon nutrient starvation, suggesting that Atg1 has a role in mating type switching independent of *RME1* expression (Supplementary Fig. S4). If the sole role of autophagy in mating type switching is to supply raw materials for protein production under starvation conditions, mating type switching should occur in the absence of autophagy upon induction in rich medium. As predicted, *RME1* overexpression in SD medium induced mating type switching in *atg1*∆ cells, as well as in wild-type cells (Fig. 7b). Therefore, these results indicated that autophagy likely supports the production of factors reliant upon Rme1-dependent transcription and thereby indirectly involved in activating their functions that are required for *MAT* inversion.

### Rme1 and a1-α2 regulate the *MAT* inversion through independent pathways

**Figure 8:**
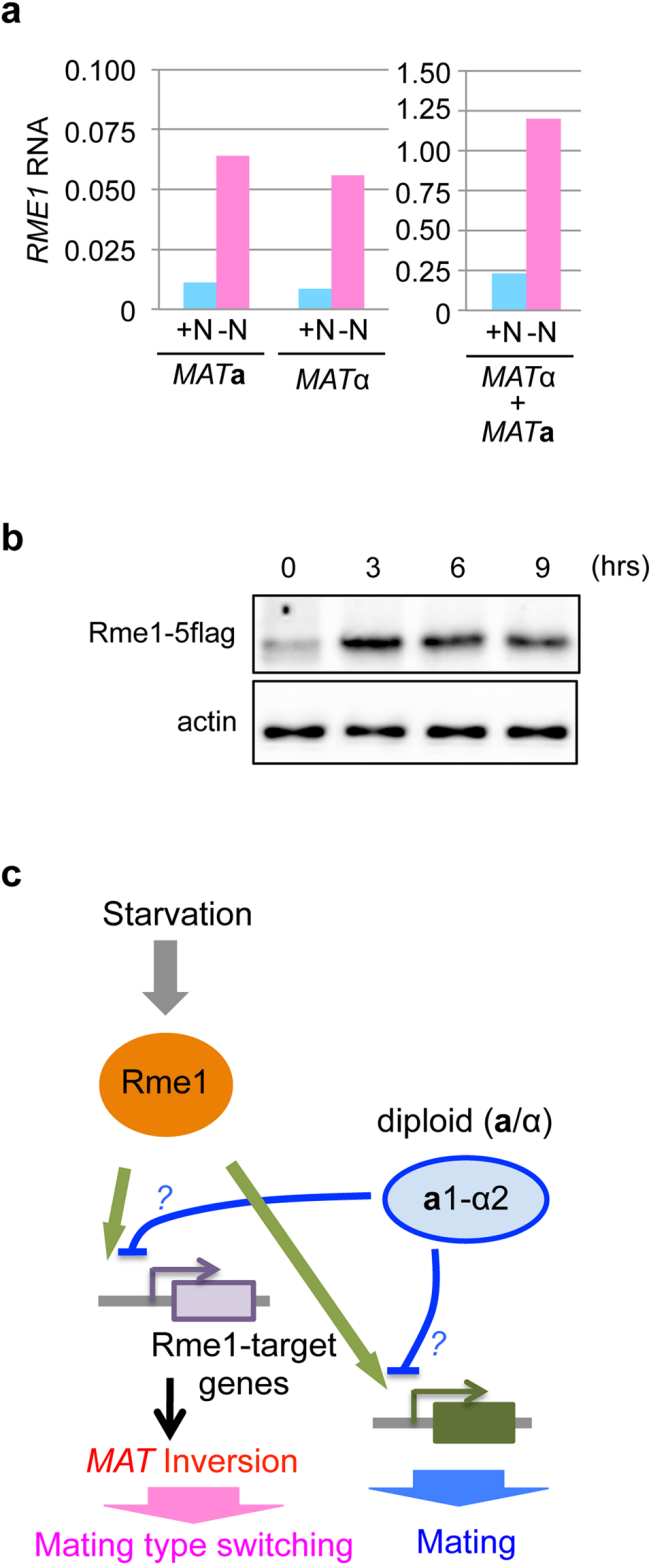
**a**1-α2 functions independently of *RME1* expression. (**a**) **a**1-α2 does not repress *RME1* mRNA levels. A(α)-type haploid cells and I(**a**)-type haploid cells in the presence or absence of *MATα* the exogenous *URA3* locus (HPH1309, HPH1311, and HPK007, respectively) were grown in YPDS (+N), then shifted to MEMA medium and incubated for 10 hrs (−N). RNA was prepared and subjected to RNA-seq analysis. Relative amount of *RME1* RNA is shown. (**b**) *RME1-5flag* cells (HPK121) were grown in YPDS (0 hr) following incubation in MEMA medium for the indicated time. Total protein was prepared and Rme1-5flag protein was detected by western blotting using an anti-flag M2 antibody. An anti-actin antibody was used as a loading control. (**c**) Model of the regulation of the mating type switching and mating in *O. polymorpha*. Full-length blots are presented in Supplementary Figure S5.

We next examined the relationship between two transcription factors, **a**1-α2, and Rme1, in mating type switching. We first performed RNA-sequencing analysis in haploid cells and the same cells mimicking a diploid state by expressing other mating type genes from an exogenous locus. We observed that *RME1* mRNA expression was induced under starvation conditions in both haploid cells and diploid-mimicking haploid cells (Fig. 8a). This result suggested that **a**1-α2 unlikely regulated *RME1* transcription. Although *RME1* mRNA levels increased 3-fold under starvation conditions, Rme1 protein levels increased only 1.5-fold (Fig. 8b), implying that Rme1-dependent functions might be regulated by other means independent of Rme1 levels, such as post-translational modifications or through the activity of other proteins.

## Discussion

Conditions under which mating type switching and/or mating occur dictate the dominant and favoured state of ploidy in vegetative cells of homothallic species. Homothallic *S. cerevisia*e strains that undergo both mating type switching and mating in rich conditions exist mostly as diploid cells, whereas in *K. lactis* and *O. polymorpha* cells that are predominantly haploid, both are induced only under nutritionally starved conditions^1^. Although *K. lactis* employs essentially the same 3-*MAT* system as *S. cerevisiae* for mating type switching, the molecular mechanisms and their regulation differ in order to allow adaption to species-specific life cycles^3,6,10^. We previously reported that the mating type in *O. polymorpha* is switched by inverting the intervening region of two *MAT* loci that reside on the same chromosome, and that homothallism in this yeast relies upon this^8^. Because an *O. polymorpha* mating type is stably maintained during vegetative growth and the switching is induced only when the environment becomes nutritionally poor^7,8^, which engages the sexual differentiation program, this process is likely coordinated with the mating process. Here, we reported that mating type switching in *O. polymorpha* was regulated by two distinct transcriptional programs: **a**1-α2 linked to the ploidy and Rme1 transmitting the starvation signal (Fig. 8c).

Determining the link between starvation and *MAT* inversion is important for understanding the sexual cycle of *O. polymorpha*. Our observation that *RME1*-overexpression induced *MAT* inversion in mitotically growing cells, which occurs only under starvation conditions in wild-type cells, suggested that the starvation signal acted through Rme1 activity. However, Rme1 is unlikely to be the sole factor responsible for inducing *MAT* inversion, because the inversion efficiency of the *rme1*∆ strain was reduced by >50% relative to that of the wild-type strain. Notably, autophagy was required for *MAT* inversion, suggesting that the essential factor(s) inducing *MAT* inversion need to be synthesized under starvation conditions. However, other transcription factor(s) responsive to starvation signals might also be involved.

Rme1 links starvation, mating, and mating type switching in *O. polymorpha* in a manner similar to that proposed for *K. lactis*; however, the composition of *MAT* loci and the molecular mechanism associated with mating type switching appear to differ between *K. lactis* and *O. polymorpha* ^6,9,10^. While KlRme1 stimulates mating type switching in two ways, one by directly binding to *MATα* in α cells and the other by activating transcription of the *KAT1* gene in **a** cells, the function of OpRme1 is most likely to activate transcription of downstream target genes. Differences in Rme1 regulation is highlighted by the involvement of **a**1-α2; whereas *KlRME1* transcription was repressed by **a**1-α2, *OpRME1* expression was not^9^. The molecular target(s) of **a**1-α2 in *O. polymorpha* that inhibit *MAT* inversion remain unknown.

The mechanisms associated with Rme1 regulation in response to starvation remains unknown. Although *OpRME1* mRNA levels were strongly induced by starvation, their protein levels were affected only moderately under our experimental conditions. Although regulation of Rme1 levels might account for the difference in Rme1 activity between rich and starvation conditions, there might also be another layer of regulation at the post-translational level, such as nuclear import and protein phosphorylation.

The event that triggers the *MAT* inversion is currently unclear. One simple scenario might be that a factor such as an endonuclease is induced by starvation to generate DNA lesions. Such genes would be expected to express only in haploid cells within a small window of time after shifting to the starvation conditions, because we did not observe significant increases in the rate of the *MAT* inversion after prolonged incubation under starvation conditions. However, we have not been successful in identifying such genes in our RNA-seq analysis. Expression profile in *rme1*∆ cells should be analysed in future. Furthermore, the delay in the timing of the *MAT* inversion in cell cycle-arrested cells under the starvation condition may suggest that an event associated with cell cycle progression contributes to triggering the *MAT* inversion in addition to the transcriptional regulations. However, this reduction might be due to the inability to induce the inversion in metaphase when sister chromatids are closely localized and homologous recombination is active. Further analyses are required to clarify these observations.

It might be rational to restrict mating type switching under conditions where mating can occur, because this will increase the population of minor mating type cells in the original population, thereby offering better chances for sexual reproduction, while avoiding energy consumption for conducting the mating type switching event in asexually reproducing conditions. Both of the haploid species, *K. lactis* and *O. polymorpha*, employ this strategy; however, the significance of condition-dependent mating type switching should be carefully examined. In *O. polymorpha*, any culture where one mating type is dominant is capable of mating with **a** or α cells at similar frequencies, suggesting that the efficiency of mating type switching is not the limiting factor for mating. In the fission yeast *S. pombe*, where mating is induced by starvation, mating type switching occurs during mitotic growth. Therefore, it is noteworthy that mating efficiency is much higher in *S. pombe* (~80%) as compared with that observed in *K. lactis* (~20%) and *O. polymorpha* (~5%). What determines the overall mating efficiency and how much impact the efficiency of mating type switching has on self-mating frequency would be subjects of future research.

## Materials and Methods

### Yeast strains and plasmids

Strains and plasmids used in this study are listed in Supplementary Table S1. Unless otherwise indicated, yeast strains were derived from CBS4329 or NCYC495 and were generated by PCR-based methods^24^. Sequences of primers used in this study are listed in Supplementary Table S2. *O. polymorpha* cells were transformed by electroporation. The *O. polymorpha TEF1* promoter was described previously^8,25^.

### Yeast growth conditions and general methods

Yeast strains were grown in YPDS or SD medium supplemented with appropriate amino acids and nucleotides^26^. All experiments were performed at 30 °C unless otherwise indicated. Mating and meiosis were induced on MEMA or NaKG plate at 30 °C^7,8^.

### Flowcytometry and microscopy

Yeast cells were prepared for flow cytometry as described^27^. DNA was stained with propidium iodide and DNA content was measured by FACSCalibur (BD Biosciences, NJ, USA). To visualize DNA, yeast cells were fixed with 70% ethanol, washed with phosphate-buffered saline (PBS), and incubated in PBS containing 1 µg/ml 4′6,-diamidino-2-phenylindole (DAPI). Images were acquired using Eclipse80i (Nicon Co., Tokyo, Japan) equipped with Cool SNAP EZ (Photometrics, AZ, USA) and driven by Meta Vue ver. 7.7.1.0 (Molecular Devices, Inc., CA, USA). Adobe Photoshop (Adobe Systems, Inc., San Jose, CA, USA) was used to mount images.

### Determination of A(α)- or I(a)-type

Orientation of the region between IR_1_ and IR_2_ [A(α) or I(**a**)-type] were determined by PCR as previously described with the primers listed in Supplementary Table S2 ^8^. For Southern blot analysis, *O. polymorpha* genomic DNA was prepared using a standard protocol. Briefly, DNA was digested with *Eco*RI restriction enzyme before electrophoresis. A standard protocol was used for blotting and hybridization. DNA probes were prepared, and detection was performed using the AlkPhos direct labeling and detection system with CDP-Star (GE Healthcare, Pittsburgh, PA, USA). Signal intensities were quantified with ImageJ version 1.47 (National Institute of Health, Bethesda, MD, USA) and Photoshop (Adobe Systems, San Jose, CA, USA) was used to mount the images.

### Semi-quantitative mating

Yeast strains of *leu1–1*, *ura3–1*, or *ade12-cr3* genotypes were grown at 30 °C in YPDS until the A_663_ was between 0.5 and 8 . Cells were washed with PBS, diluted to A_663_ = 1.0, and a 10-µL cell suspension of the two strains was mixed on a nitrocellulose-membrane filter that was placed on a MEMA or NaKG plate and incubated for 20–30 hrs at 30 °C. Cells were resuspended in PBS, and dilutions were plated on SD plates supplemented with leucine or uracil and adenine or on unsupplemented SD plates that were incubated for 2 days at 37 °C. The mating percentage was calculated as the number of colonies on unsupplemented plates divided by the number on adenine and leucine- or uracil-supplemented plates (i.e., whichever had fewer colonies).

### RNA analysis

Total RNA was isolated from *O. polymorpha* as previously described, treated with DNase I, and then further purified using the RNeasy Plus kit or RNeasy MinElute Cleanup Kit (Qiagen, Valencia, CA, USA). A total of 800 ng-1 µg RNA was used to synthesize cDNA with SuperScriptIII (Invitrogen, Carlsbad, CA, USA) or Reverse Tra Ace qPCR RT Master Mix (Toyobo Co., Ltd., Osaka, Japan) according to the manufacturer’s protocol, and a 0.1–1 µl cDNA reaction mixture was used in a qPCR reaction with the primers listed in Supplementary Table S2.

RNAs prepared from wild type **a**, α, and **a** cells carrying *MATα* were sequenced using HiSeq system (Illumina Inc., San Diego, CA, USA). The cDNA libraries were prepared with a TruSeq DNA Sample Preparation v2 Kit (Illumina Inc.) according to the manufacturer’s protocol. The reference sequence contains only ORF sequences of *O. polymorpha* based on The JGI Genome Portal^28–30^. Supplementary information Dataset 1 shows the result of gene expression analysis.

### Data availability

The datasets generated during and/or analysed during the current study are available from the corresponding author on reasonable request.

## Electronic supplementary material

Supplementary information

Dataset 1

Supplementary Table S1

Supplementary Table S2
