# Supplementary material for: Regulation of mating type switching by the mating type genes and RME1 in Ogataea polymorpha
Source: Sci Rep. 2017 Nov 24;7:16318. doi: 10.1038/s41598-017-16284-7 (PMC5701183; doi:10.1038/s41598-017-16284-7)

Supplementary information

Regulation of mating type switching by the mating type genes and *RME1* in *Ogataea polymorpha*

Katsuyoshi Yamamoto[1], Thi N. M. Tran[2], Kaoru Takegawa[2], Yoshinobu Kaneko[1] and Hiromi Maekawa[1,2]*

1 Graduate School of Engineering, Osaka University, Osaka, Japan
2 Faculty of Agriculture, Kyushu University, Fukuoka, Japan

*Corresponding author
Hiromi MAEKAWA
Centre for Promotion of International Education and Research
Faculty of Agriculture
Kyushu University
6-10-1 Hakozaki, Higashi-ku, Fukuoka 812-8581, JAPAN
Phone: +81 92-642-2126
Fax: +81 92-642-2126
E-mail: hmaekawa@agr.kyushu-u.ac.jp

Supplementary figure S1 Mating type genes were in rich and starvation conditions

(a) Unswitchable wild type A(α)-type and I(**a**)-type haploid cells (HPH1172 and HPH1160) were grown in YPDS medium and transferred to NaKG medium and incubated for the indicated time. RNA were prepared and subjected to qPCR analysis of **a***1*, **a***2* genes for HPH1172, and *α1*, *α2* genes for HPH1160. RNA levels were normalized to that of *ACT1* RNA. Shown are the averages of three independent PCR reactions. Error bars indicate SD. (b, c) A(α)-type haploid cells and I(**a**)-type haploid cells in the presence or absence of *MATα* the exogenous *URA3* locus (HPH1309, HPH1311, and HPK007, respectively) were grown in YPDS (+N), then shifted to MEMA medium and incubated for 10 hrs (-N). RNA was prepared and subjected to RNA-seq analysis. Relative amount of α*2 m*RNA (**a**) and **a***1* mRNA (**b**) is shown.

Supplementary figure S2 *STE4* and *STE18* mRNA were induced by starvation

A(α)-type haploid cells and I(**a**)-type haploid cells in the presence or absence of MATα the exogenous *URA3* locus (HPH1309, HPH1311, and HPK007, respectively) were grown in YPDS (+N), then shifted to MEMA medium and incubated for 10 hrs (-N). RNA was prepared and subjected to RNA-seq analysis. Relative amount of *STE4 m*RNA (**a**) and *STE18* mRNA (**b**) is shown.

Supplementary figure S3 Mating is severely reduced in *rme1*Δ cells.

Wild type and *rme1*Δ strains of *ura3-1* genotypes (HPH719 and HPK078, respectively) were combined with and inversion-deficient stable **a** and α cells of *ade12-cr3* genotype (HPH1160 and HPH1172) on MEMA mating medium and incubated at 30°C. After 15 h, cells were spread on SD plates to select for Ade^+Ura^+ diploids. Colony number was counted after a 2-days incubation at 37°C. The average of three independent matings is shown. Error bars indicate SD.

Supplementary figure S4 Induction of *RME1* expression is not dependent on Atg1.

Wild type cells (BY21401) and *atg1*Δ cells (HPH1620) were grown in YPDS medium and transferred to NaKG medium and incubated for 4 hrs. RNA were prepared and subjected to qPCR analysis for *RME1*. RNA levels were normalized to

that of *ACT1* RNA. Shown are the averages of three independent PCR reactions. Error bars indicate SD.

Supplementary figure S5 Full-length gels and blots of Figures
The areas that appear in Figure are marked with rectangles.

Supplementary table S1 Yeast strains and plasmids

Supplementary table S2 Primers

Supplementary table S3 Gene expression data in RNA-seq analysis

Supplementary figure S1

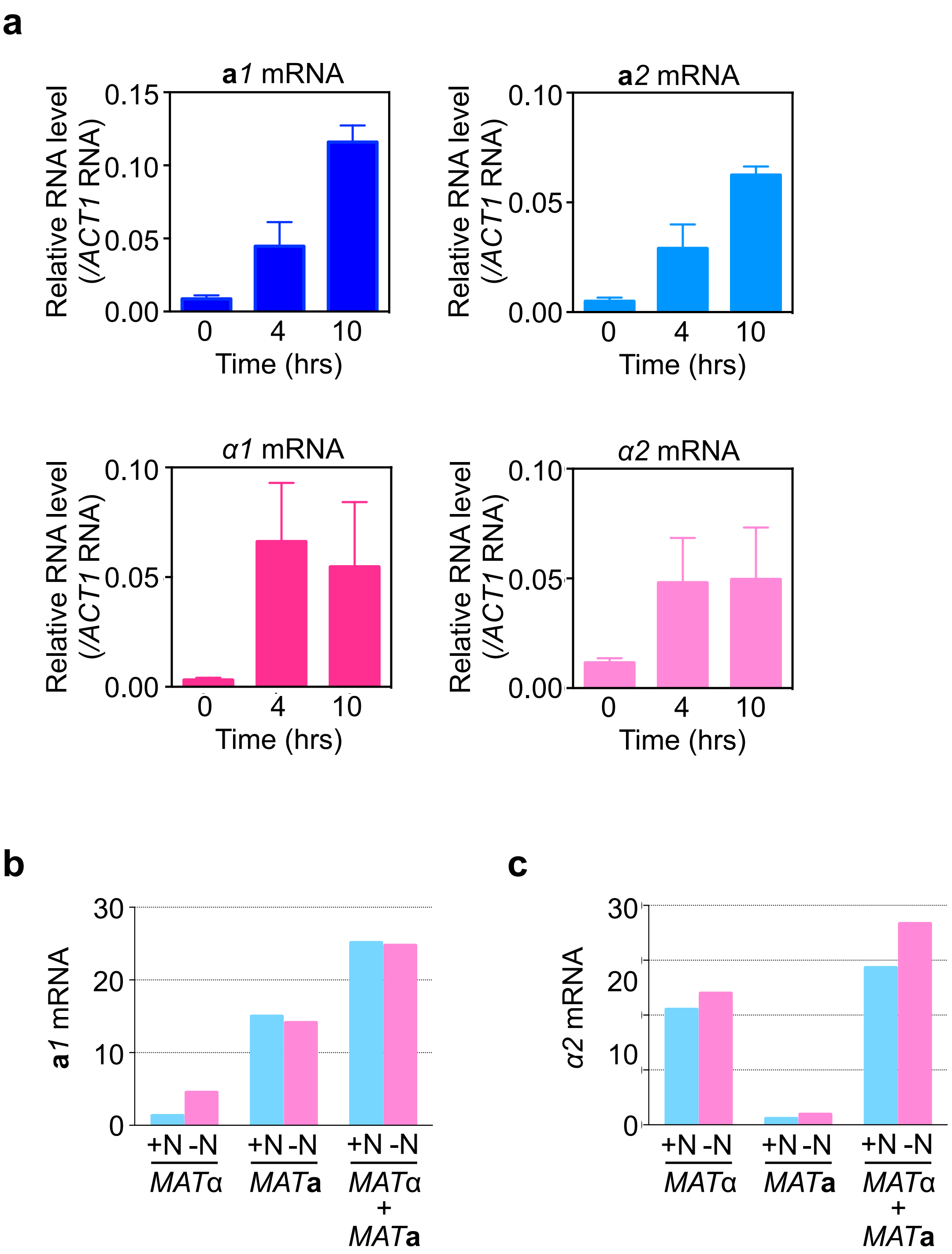

Supplementary figure S2

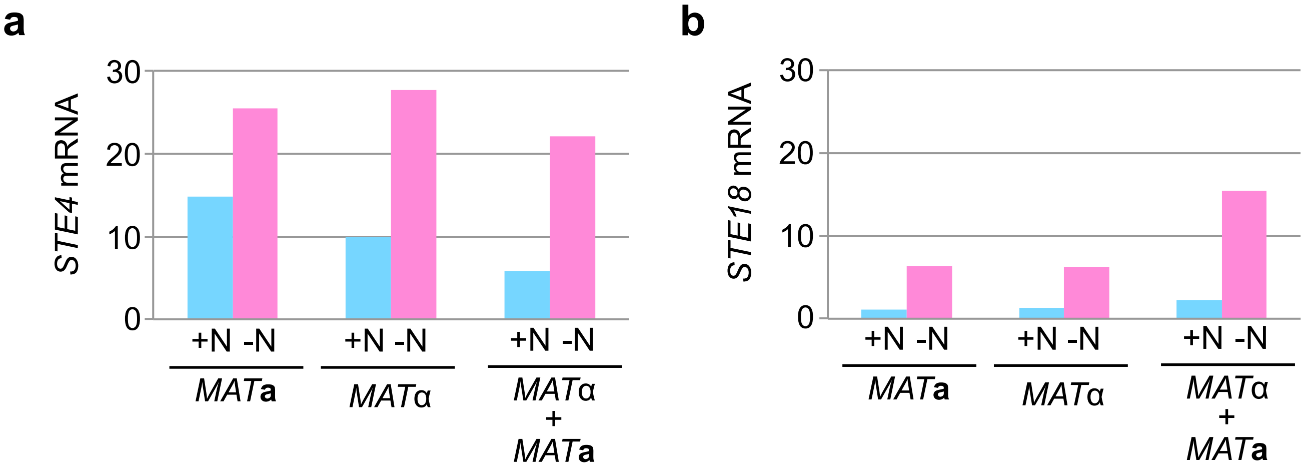

Supplementary figure S3

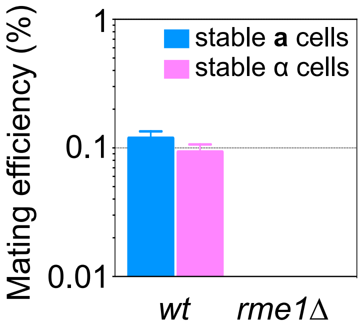

Supplementary figure S4

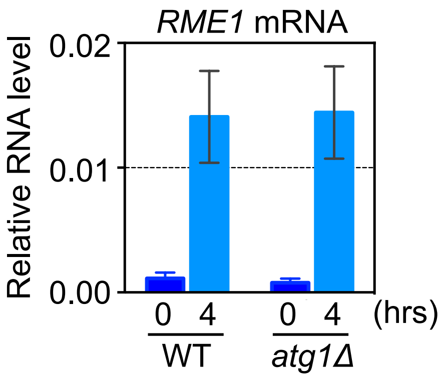

Supplementary figure S5 Full-length gels and blots in Figure 1-8

Figure 1d

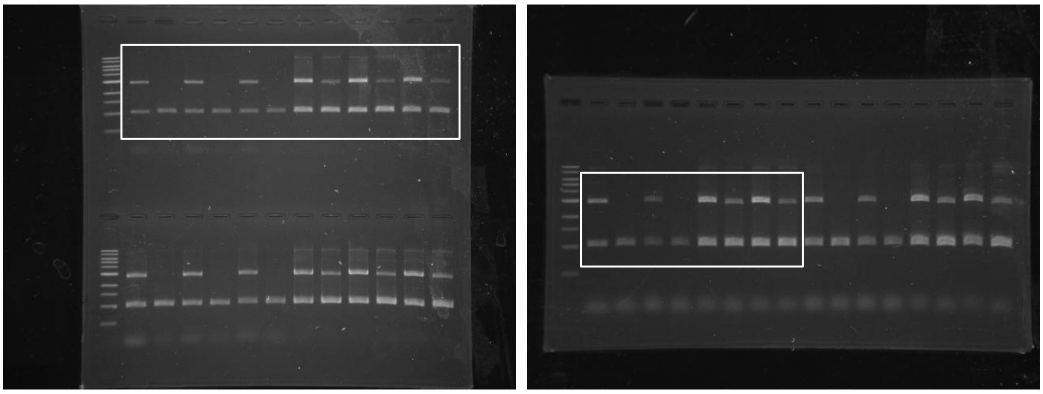

Figure 2a

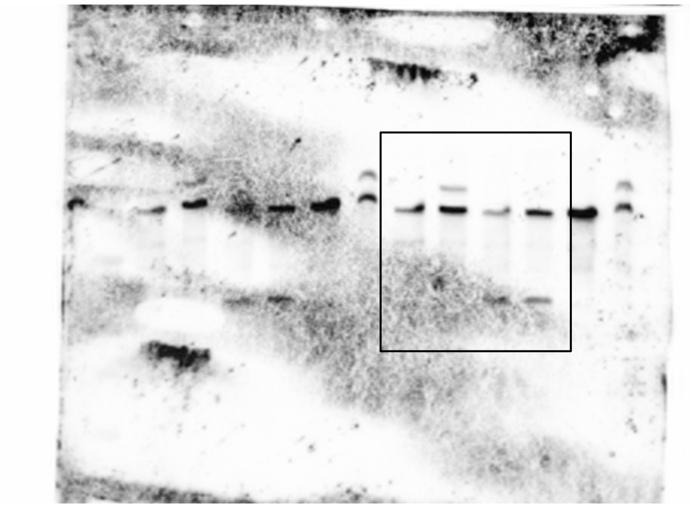

Figure 2b

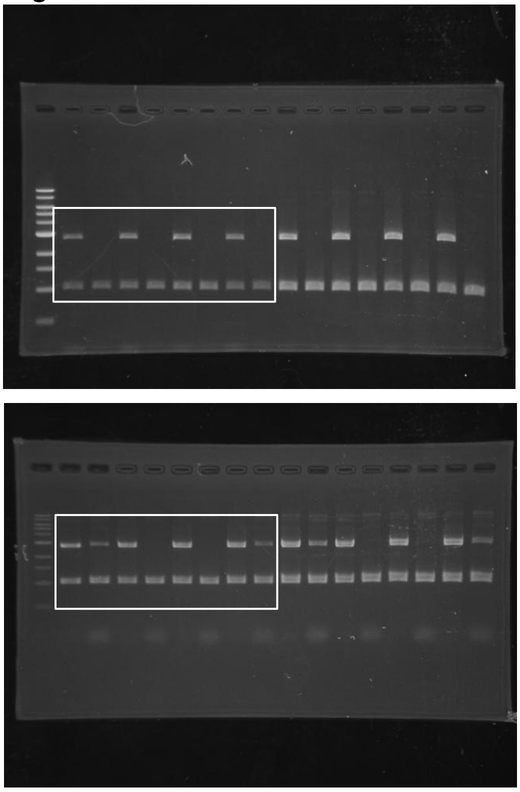

Figure 2c

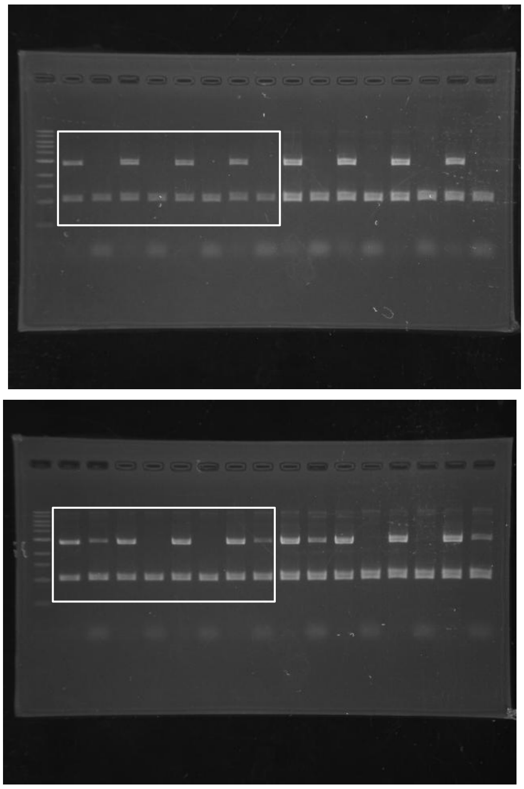

Supplementary figure S5 Full-length gels and blots in Figure 1-8

Figure 3b

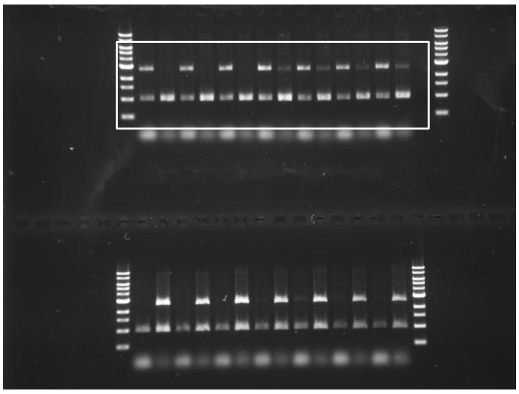

Figure 3c

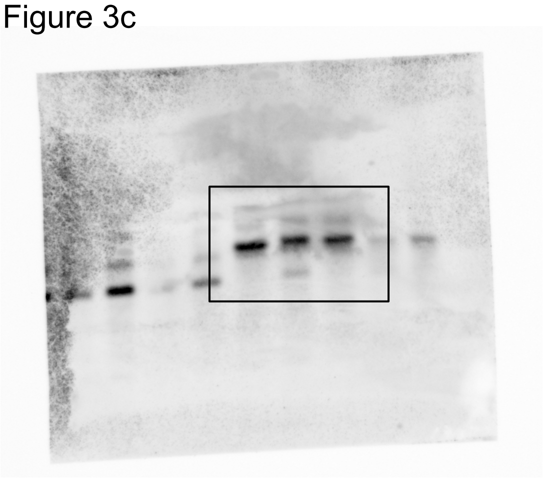

Figure 4a

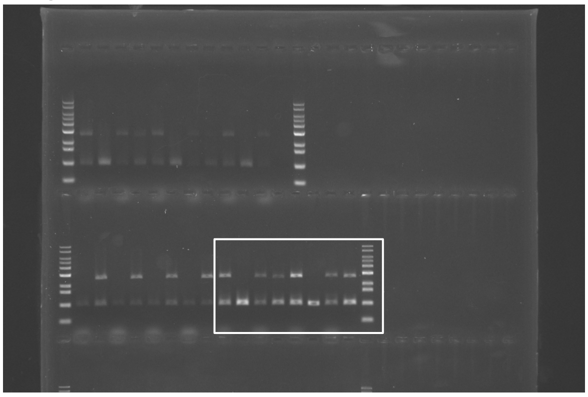

Figure 4b

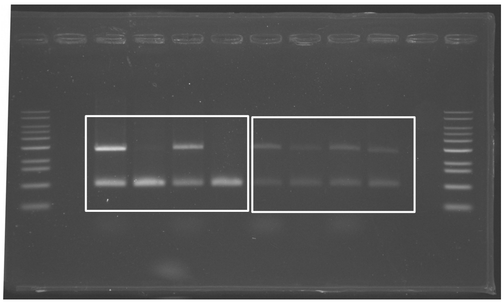

Figure 5d

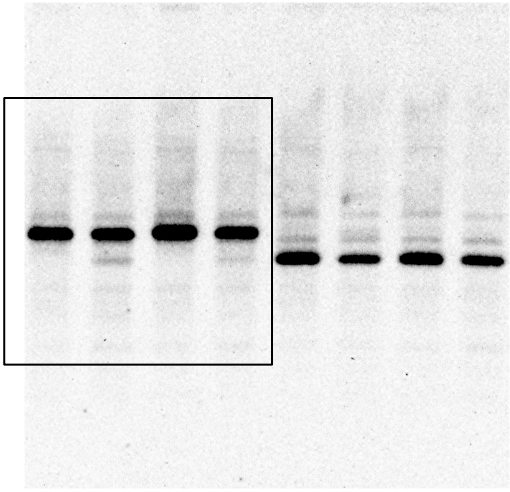

Supplementary figure S5 Full-length gels and blots in Figure 1-8

Figure 6a

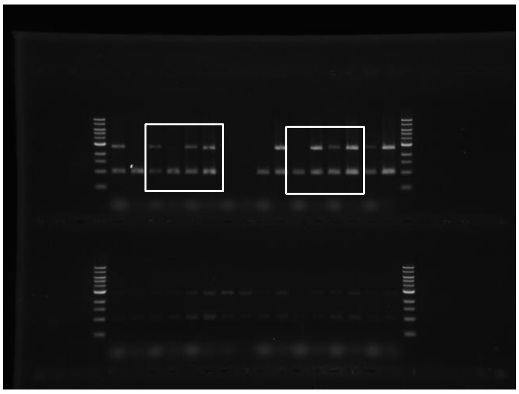

Figure 6b

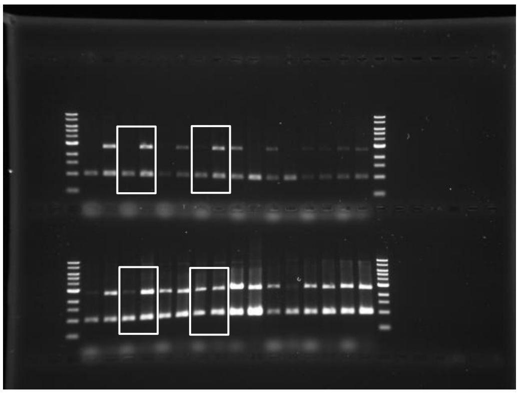

Figure 6d

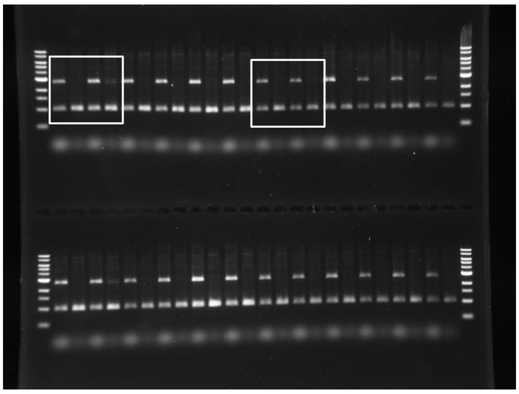

Figure 7a

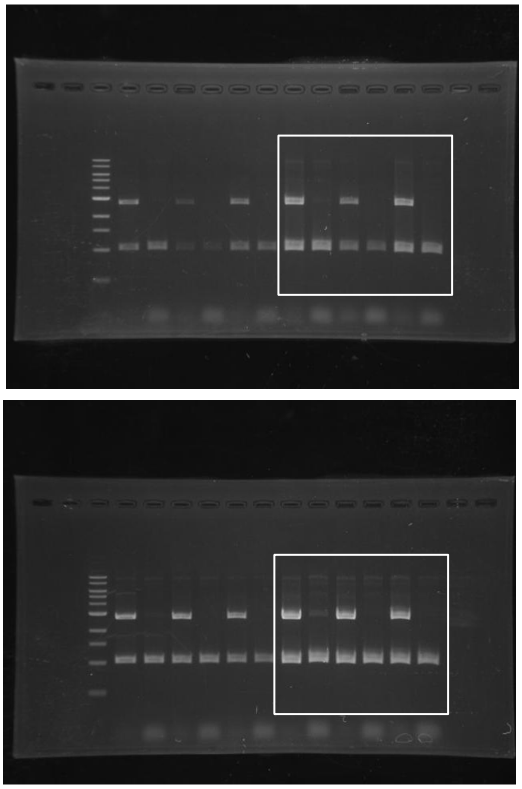

Figure 7b

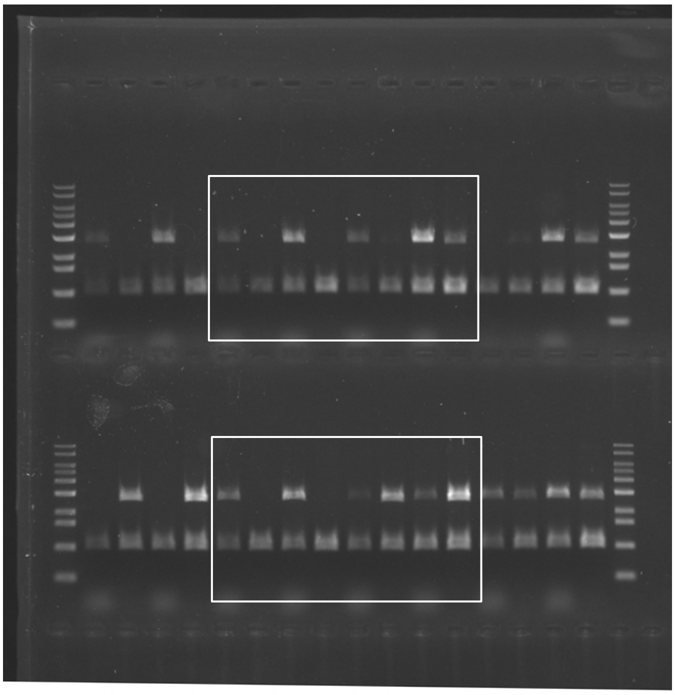

Supplementary figure S5 Full-length gels and blots in Figure 1-8

Figure 8b

Rme1-5flag

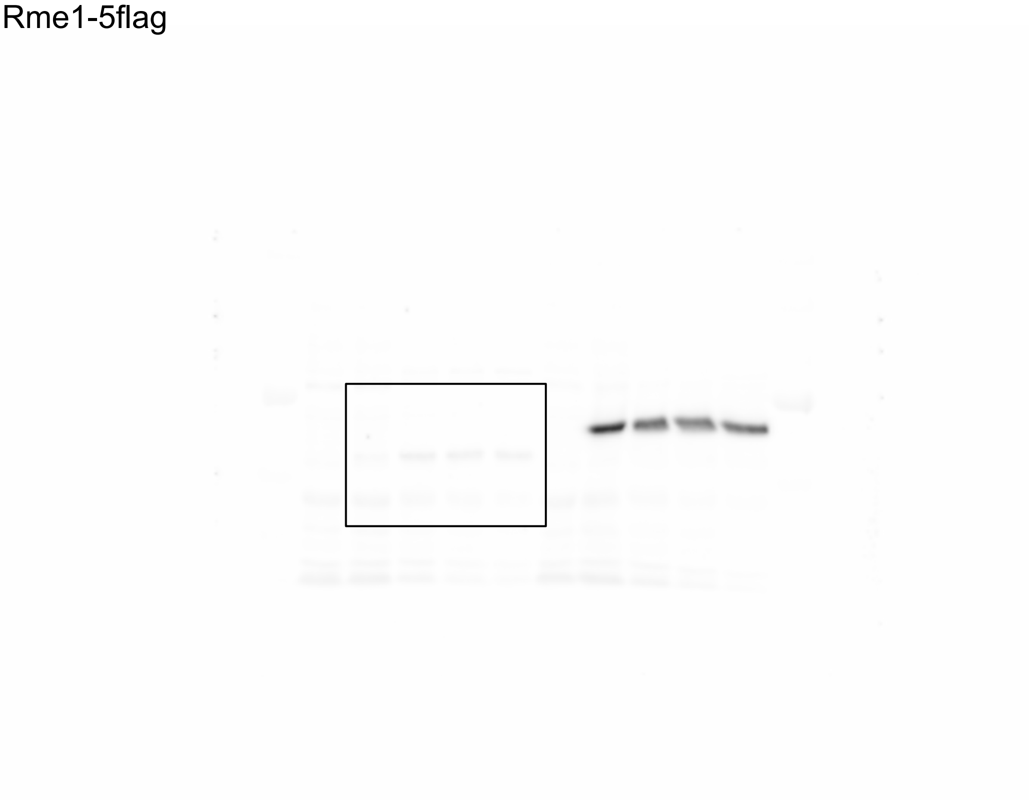

actin

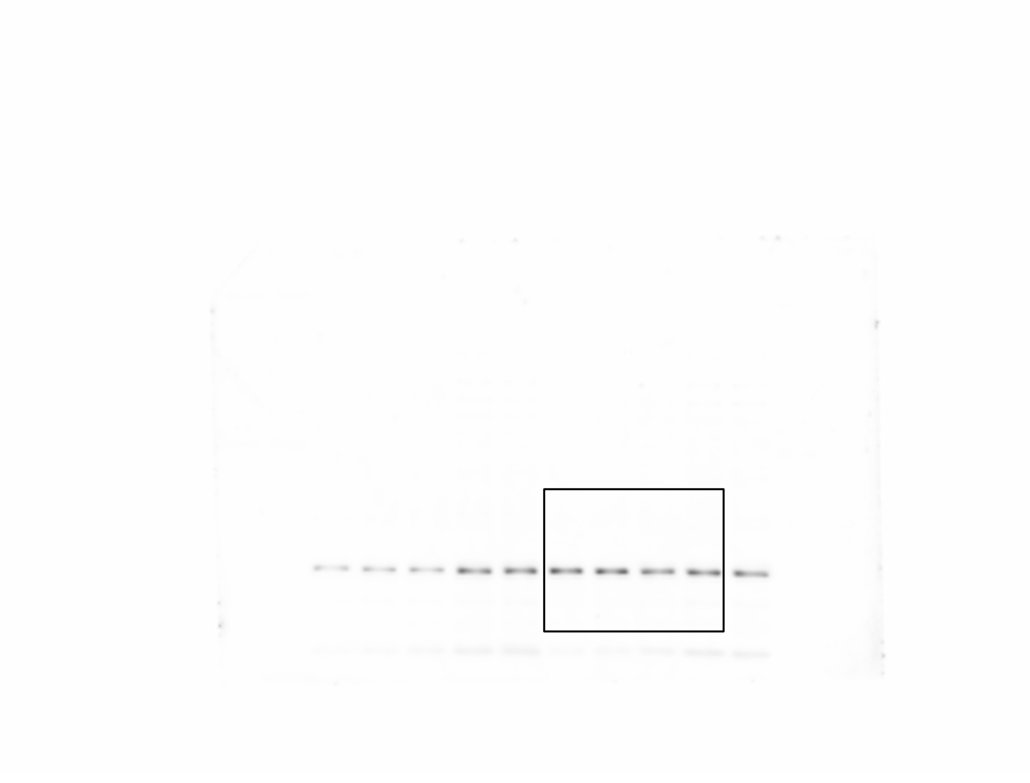

Supplementary figure S5 Full-length gels and blots in Figure 1-8

Figure 5d

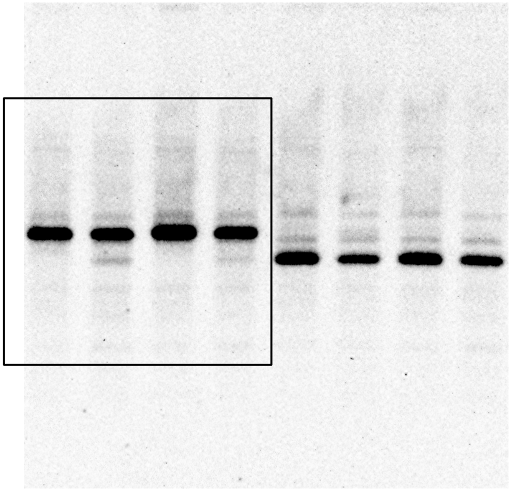

Supplement: Supplementary file 1 — Supplementary information [file 41598_2017_16284_MOESM1_ESM.pdf]
