# Supplementary material for: Regulation of mating type switching by the mating type genes and RME1 in Ogataea polymorpha
Source: Sci Rep. 2017 Nov 24;7:16318. doi: 10.1038/s41598-017-16284-7 (PMC5701183; doi:10.1038/s41598-017-16284-7)
Supplement: Supplementary file 3 — Supplementary Table S1 [file 41598_2017_16284_MOESM3_ESM.pdf]

Supplementary Table S1 Yeast strains and plasmids

Name	Genotype/construction	Source
**Yeast strains**		
BY4330	*ura3-1*	NBRP-Yeast
BY4330	*ade11-1*	NBRP-Yeast
BY21401	CBS4329, Type strain	NBRP-Yeast
HPH22	*leu1-1*	ref. 8
HPH47	*ku80Δ::hphNT1 ura3-1*	This study
HPH466	A(α)	This study
HPH553	*ste2Δ::natNT2 leu1-1*	ref. 8
HPH581	*ste3Δ::hphNT1 leu1-1*	ref. 8
HPH719	*ura3-1*	This study
HPH848	A(α)	This study
HPH922	*a2Δ::natNT2 ura3-1*	This study
HPH964	I(a) *IR2Δ::3mcherry::natNT2*/A(α) *ura3-1*/+ +/*leu1-1*	This study
HPH1047	A(α)	This study
HPH1050	I(**a**)	This study
HPH1162	A(α) *IR2Δmat-aΔ::3mcherry::natNT2 leu1-1 ade12-cr3*	This study
HPH1170	I(**a**) *IR2Δmat-αΔ::3mcherry::natNT2 ura3-1*	This study
HPH1172	I(**a**) *IR2Δmat-αΔ::3mcherry::natNT2 ade12-cr3*	This study
HPH1174	I(**a**) *IR2Δmat-αΔ::3mcherry::natNT2 ade12-cr3 ura3-1*	This study
HPH1201	A(α) *ura3-1*::pHM876	This study
HPH1255	I(**a**) *a2Δ::natNT2*	This study
HPH1267	*ste4Δ::hphNT1 URA3::*pHM901	This study
HPH1268	*ste4Δ::hphNT1*	This study
HPH1309	A(α) *IR2Δmat-**a**Δ::natMT2* (stable *MATα*)	This study
HPH1311	I(**a**) *IR2Δmat-αΔ::natMT2* (stable *MAT***a**)	This study
HPH1378	CBS4732 *ku80Δ::zeo*	This study
HPH1535	*ura3-1 MET3::*pHM965	This study
HPH1556	*atg13Δ::natNT2*	This study
HPH1561	*atg1Δ::natNT2*	This study
HPH1620	CBS4732 *atg1Δ::natNT2*	This study
HPH1696	CBS4732 *ku80Δ::zeo ste2Δ::natNT2 ste3Δ::hphNT1*	This study
HPH1699	A(α) *IR2Δmat-aΔ::3mcherry::natNT2 leu1-1 ura3-1*::pHM964	This study
HPH1702	I(**a**) *IR2Δmat-αΔ::3mcherry::natNT2 ade12-cr3 ura3-1*::pHM961	This study
HPK007	I(**a**) *ΔIR2Δmat-α::3mcherry::natMT2 ura3-1*::pHM875	This study
HPK021	*rme1Δ::natNT2 ku80Δ::hphNT1 ura3-1*	This study
HPK011	I(**a**) *ura3-1*::pHM875	This study
HPK072	A(α) *mat-αΔ::natNT2*	This study
HPK073	I(**a**) **a***1Δ::natNT2*	This study

HPK078	*rme1Δ::natNT2 ura3-1*	This study
HPK084	I(**a**) *URA3::*pHM961	This study
HPK085	I(**a**) *URA3::*pHM962	This study
HPK092	A(α) *URA3::*pHM964	This study
HPK093	A(α) *URA3::*pHM963	This study
HPK121	*RME1-5FLAG-hphNT1*	This study
HPK141	A(α)	This study
HPK187	CBS4732 *rme1Δ::natNT2*	This study
HPK189	CBS4732 *rme1Δ::natNT2*	This study
**Plasmids**		
pHM741	pFA6a-kanMX6 carrying *KU80*	This study
pHM874	*HpURA3-hphNT1*-based integration plasmid carrying P_{TEF1}	This study
pHM875	*HpURA3*-based integration plasmid carrying *MATα*	This study
pHM876	*HpURA3*-based integration plasmid carrying *MAT***a**	This study
pHM960	*HpURA3*-hphNT1-based integration plasmid carrying P_{TEF1}-*RME1*	This study
pHM961	*HpURA3*-based integration plasmid carrying *MATα*(*α1Δ*)	This study
pHM962	*HpURA3*-based integration plasmid carrying *MAT*α(*α2Δ*)	This study
pHM963	*HpURA3*-based integration plasmid carrying *MAT***a**(**a***1Δ*)	This study
pHM964	*HpURA3*-based integration plasmid carrying *MAT***a**(**a**2*Δ*)	This study
pHM901	*HpURA3-kanMX6*-based integration plasmid carrying *STE4*	This study
