# Supplementary material for: Regulation of mating type switching by the mating type genes and RME1 in Ogataea polymorpha
Source: Sci Rep. 2017 Nov 24;7:16318. doi: 10.1038/s41598-017-16284-7 (PMC5701183; doi:10.1038/s41598-017-16284-7)
Supplement: Supplementary file 4 — Supplementary Table S2 [file 41598_2017_16284_MOESM4_ESM.pdf]

Supplementary table S2. Primers

Name	Sequences	Purpose
a1-F	TGACTACCAGCCTGATAGGCAAC	Real-Time PCR for **a***1*
a1-R	GGAAGCAAGGTCCCCTTCTC	Real-Time PCR for **a***1*
a1-S1	GGGTTAAGCAACACTATAGGAAGCTCTTGGATGACTGGGAGGCGGCTATCCGTACGCTGCAGGTCGAC	constuction of **a***1Δ*
a1-S2	TTAGAAAATTCAGCTACAGAGATGAAGAATCCTAGAATGCTTCAATCTTAATCGATGAATTCGAGCTCG	constuction of **a***1Δ*
a2-F	ACACTAACAGACGACGGAGA	Real-Time PCR for **a***2*
a2-R	GAAGGGCGTAGCAAACACTC	Real-Time PCR for **a***2*
a2-S1	AGAGATCAAAGATAGATCTTCAGAAGTCCAAATCAATAACGAAAATATCGTAGTCAAATGCGTACGCTGCAGGTCGAC	constuction of **a***2Δ*
act1-4	ACAACGAGTTGAGAGTTGCG	Real-Time PCR for *ACT1*
act1-5	GGTCGTTCTACCAGACGAGT	Real-Time PCR for *ACT1*
alpha1-F	CAGATGCGTGGAATCGTGAA	Real-Time PCR for *α1*
alpha1-R	TTGAAAACGGTTCCTTGCTCTT	Real-Time PCR for *α1*
alpha1-S1	TTGAATCAAGATACACGCTGCTCTCCCAATGATGTAGTATAATAAAAATGCGTACGCTGCAGGTCGAC	constuction of *α1Δ*
alpha1-S2	AATATTGTGAACTGCATTCCTGAAATACATCCTCGACTACTATATGGGACATCGATGAATTCGAGCTCG	constuction of *α1Δ*
alpha2-F	AAATACACACGGGATTGCC	Real-Time PCR for *α2*
alpha2-R	AATCGTTTGGAGCGCTTCTC	Real-Time PCR for *α2*
alpha2-S1	TAACAAAAGGTTATCACACATACTGCAAGCTCCCGGTCAGGGAGTCCCATGGATGATATGCGTACGCTGCAGGTCGAC	constuction of *α2Δ*
alpha2-S2	AAAGAACATGAAACATCATTTTTATTATACTACATCATTGGGAGAGCAGCGTGTATCTTGATCGATGAATTCGAGCTCG	constuction of *α2Δ, IR2Δmat-αΔ*
ATG1-S1F	CAAGCACCACATATGTGTGC	constuction of *atg1Δ*
ATG1-S1R	GTCGACCTGCAGCGTACGCATCGGGTCGACTAAGCCTTAC	constuction of *atg1Δ*
ATG1-S2F	CGAGCTCGAATTCATCGATACAGGGAGTAAGAAGTTAGG	constuction of *atg1Δ*
ATG1-S2R	AGTCGCTGTAGTTGCTGCTG	constuction of *atg1Δ*
ATG13-S1F	CTGGGACAGTTCCATGTTCC	constuction of *atg13Δ*
ATG13-S1R	GTCGACCTGCAGCGTACGCATTGCTGATTAATTTACAATAACAAGCC	constuction of *atg13Δ*
ATG13-S2F	CGAGCTCGAATTCATCGATTAGCCAAGAATAATCAGGAG	constuction of *atg13Δ*
ATG13-S2R	GCAGAACTTGGACTCTGTCC	constuction of *atg13Δ*
CDC28-11	AACACAACAACCGCGTAGTG	amplify *CDC28* fragment
CDC28-5	CTCCATCTTTGTGCTGTTGC	amplify *CDC28* fragment
contig66-LS-S2	GTATCTCCACCTGCTGTTCGATCGATGAATTCGAGCTCG	constuction of **I**(a) *IR2Δ*
contig66L-S1	TACATGTCATGTGCTCTATGTAGGGAAAATGCACGGTAGCAGCTCTGCGGGGTGCATGGTCGTACGCTGCAGGTCGAC	constuction of **I**(a) *IR2Δ*, *IR2Δmat-αΔ, IR2Δmat-***a***Δ*

N-alpha2-S2	TAACAAAAGGTTATCACACATACTGCAAGCTCCCGGTCAGGGAGTCCCATGGATGATATGATCGATGAATTCGAGCTCG	constuction of *IR2Δmat*-**a***Δ*
primer_A	AGGAACAGGTTCAGTACTGG	PCR a and PCR α
primer_B	TTAAACAAGGTAGCACCGAAAAA	PCR α
primer_C	ATAAGTACTCACAATCGAGGC	PCR a
RME1-1	GCAGCAGCAAGAGCCAAACT	Real-Time PCR for *RME1*
RME1-2	TTGCGGGAAGCCATTAGC	Real-Time PCR for *RME1*
RME1-S1	ACTTTACGCTCGTGACCTTTAAATTGCATTCTGAAAGTGCGCTTTCGTAGACGTGGGACACGTACGCTGCAGGTCGAC	constuction of *rme1Δ*
RME1-S2	ACCGCTCTGGACAACGAATAAAATCCACGGAAGTCGGAGTGAACATTAAATATTAGAGACATCGATGAATTCGAGCTCG	constuction of *rme1Δ*
Sa2-S2	CACTATTACGTAATCCAGTGAAACGCTTTCGGTGATATAATAGTTACCCGCTCTGGATTAATCGATGAATTCGAGCTCG	constuction of **a***2Δ*
STE2-S1	ATCCCTCCCAGCATATAAAGTGCAAGGCTCGACACCTCTATTTACACAATCTTCACAATGCGTACGCTGCAGGTCGAC	constuction of *ste2Δ*
STE2-S2	CTCGCTGACTTTTAAGTATTAAATTTTGTTATGGACGATCGAAAAACCGATAATTAATTAATCGATGAATTCGAGCTCG	constuction of *ste2Δ*
STE3-S1	AGCTGACGGGAAAATTATATAAAGCGAGCGGCAGGGAGACAGCAAAATGCATACAGAATGCGTACGCTGCAGGTCGAC	constuction of *ste3Δ*
STE3-S2	CCTGGCCTTTTTTAAATCTGCTTTGTAAGTTAGTCTTAGCTCAATCGAAATAAGTAATTAATCGATGAATTCGAGCTCG	constuction of *ste3Δ*
STE4-S1	GCTGTGCAGCGGTGAGGTAGCACAAGATGGAGAGTTGCTGAATCTTGAAAGATTATCATGCGTACGCTGCAGGTCGAC	constuction of *ste4Δ*
STE4-S2	GCGGGCAGAGTAGATGTAGATGTATTTTACGAGTGTATACAATACATGCGCTTATTACTAATCGATGAATTCGAGCTCG	constuction of *ste4Δ*
